# Status of tetanus prevention following injuries: a descriptive study from Sandun Town, Hangzhou

**DOI:** 10.3389/fpubh.2026.1799196

**Published:** 2026-04-07

**Authors:** Ju Sun, Qilong Wang, Da Sun, Hongfei Qian, Yuxiang Huang, Anqi Liu, Lvzhao Liao

**Affiliations:** 1 Department of Chinese Medicine Development, The First Affiliated Hospital of Zhejiang Chinese Medical University, Hangzhou, Zhejiang, China; 2 Emergency Department, Zhejiang Hospital, Hangzhou, Zhejiang, China; 3 Second School of Clinical Medicine, Zhejiang Chinese Medical University, Hangzhou, Zhejiang, China

**Keywords:** emergency department, tetanus, injuries, prophylaxis, communicable disease

## Abstract

**Objective:**

To describe tetanus vaccination practices and injury characteristics in Sandun Town, Hangzhou during February 2024 to January 2025.

**Methods:**

A retrospective cross-sectional study was conducted using data from trauma and animal-injury patients who received tetanus vaccination in Sandun Town, Hangzhou from February 2024 to January 2025. Demographic characteristics, injury profiles, vaccination status, and relationships with season/temperature were analyzed.

**Results:**

Total of 3,174 patients were initially identified, and 2,825 were included in the final analysis after applying exclusion criteria. Among them, animal-induced injuries accounted for 1,029 cases (36.4%), traffic accident injuries for 943 cases (33.4%), cutting injuries for 374 cases (13.2%), blunt force injuries for 242 cases (8.6%), and other causes for 237 cases (8.4%). Males predominated in all trauma categories except animal-induced injuries, where females were the majority (53.3%). Employees/workers were the predominant occupational group across all categories (56.4–76.4%). The upper limb was the most common injury site across all categories (56.7–74.1%). High-risk wounds were observed in 88.2–98.9% of patients. The use of passive immunizing agents was generally low (2.1–10.4%), with the highest rate in animal-induced injuries.

**Conclusion:**

Following the implementation of China’s 2024 non-neonatal tetanus guideline, the treatment of external injuries is becoming more standardized. However, gaps persist compared with developed countries, mainly reflected in the underutilization of passive immunizing agents for high-risk wounds and inconsistent application of guidelines. Targeted education for students, migrant workers, and pet owners-particularly during warmer months-and continued training for healthcare personnel are needed.

## 1 Introduction

Tetanus is a severe and potentially fatal disease caused by *Clostridium tetani*. The pathogen typically enters the human body through broken skin or wounds and subsequently releases tetanus neurotoxin. This toxin enters inhibitory interneurons and blocks the release of glycine or *γ*-aminobutyric acid (GABA), leading to spastic paralysis. Classic symptoms include muscle contractures and persistent spasms of skeletal muscles (1). Severe cases may experience laryngospasm, asphyxia, pulmonary infections, or even organ failure, any of which can be fatal. The case-fatality rate for severe tetanus approaches 100%. Even with prompt, strict, and standardized medical intervention, the global mortality rate for tetanus patients remains between 30 and 50% (2). Due to the persistent presence of *C. tetani* spores in the environment, the possibility of eradicating tetanus is virtually zero (3). However, with the widespread application of tetanus vaccines and immunoglobulins, along with coverage by planned immunization programs, tetanus has become very rare in developed countries in recent years. In low- and middle-income countries and regions, particularly in South Asia and Africa, tetanus remains a serious public health problem. A meta-analysis indicated that the mortality rate among tetanus patients in Africa is approximately 43.2% (95% CI [36.9, 49.5%]) (4).

In China, thanks to the implementation and improvement of planned immunization, neonatal tetanus has been eliminated (5). Regarding non-neonatal tetanus, data show that in 1990 alone, China reported 37,160 tetanus cases, resulting in 12,439 deaths (6). Two decades later, from 2010 to 2017, a total of 3,992 tetanus cases were reported nationwide, with 272 deaths (7). Although this represents a significant decline compared to historical data, a substantial gap persists when compared to developed countries such as the United States, particularly in terms of medical burden. Studies have indicated that the disease burden of post-traumatic tetanus in China is heavy, with an incidence rate dozens of times higher than that in the United States and European countries (8–10), posing a serious threat to public life and property. This has prompted reforms in tetanus disease management in China.

Previously, only tetanus passive immunizing agents were used for prevention in post-traumatic patients. A study from Guizhou Province pointed out that from 2018 to 2022, the total number of tetanus vaccine doses administered in the entire province was only 4,972 (11). Tetanus vaccine was not prioritized, and prevention relied largely on tetanus immunoglobulins, whose safety and protective efficacy are incomparable to those of the tetanus vaccine. In the past, healthcare personnel typically followed the 2016 Chinese guidelines for the diagnosis and treatment of non-neonatal tetanus, which primarily involved administering passive immunizing agents-namely human tetanus immunoglobulin or equine tetanus immunoglobulin-for general trauma, without emphasizing the role of the tetanus vaccine. The situation changed with the release of the 2024 Chinese guidelines for the diagnosis and treatment of non-neonatal tetanus (12). For the prevention of tetanus in patients with general trauma or animal-induced injuries, the new guidelines emphasize assessing wound risk and immunization history, and standardizing the use of both tetanus vaccine and tetanus passive immunizing agents. The 2024 guidelines simplify the risk classification, condensing the WHO-recommended three-level wound risk into two categories-low risk and high risk-making clinical operations more straightforward. Furthermore, they place greater emphasis on active immunization rather than passive immunization. A fundamental conceptual advancement in the new guidelines is the establishment of vaccine-based active prevention as the core strategy, thereby elevating the priority of the tetanus vaccine over the subsequent use of tetanus immunoglobulins. This makes the management of trauma and animal-induced injury patients more standardized.

Zhejiang hospital has been implementing tetanus vaccination since February 2024, which is the only medical institution providing tetanus vaccination in Sandun Town, Hangzhou, the data collected from our emergency department are broadly representative of the tetanus vaccination practices among trauma and animal-injury patients in this region. This report aims to present the one-year data on tetanus vaccination in Sandun Town, Hangzhou.

## 2 Materials and methods

### 2.1 Data source and study design

A retrospective cross-sectional study was conducted, enrolling trauma patients and animal-induced injury patients who received tetanus vaccination at the Emergency Department of Zhejiang Hospital between February 2024 and January 2025. Clinical data were sourced from the hospital’s electronic medical record (EMR) system in the Emergency Department. Data were extracted from the EMR system by emergency physicians using a standardized data collection form. Patients’ history of prior tetanus vaccination was determined through patient self-reporting and by verifying historical vaccination records within the regional health information system. For variables such as wound classification and exposure risk, the primary reference was the information documented in the medical records by the attending emergency physician.

### 2.2 Inclusion criteria included

#### 2.2.1 Inclusion criteria

(1) No missing clinical data for the patient. (2) The patient received tetanus immunoprophylaxis in the Emergency Department of Zhejiang Hospital. (3) The patient had no cognitive impairment.

#### 2.2.2 Exclusion criteria

Incomplete collection of necessary information or refusal to receive prophylactic immunization.

### 2.3 Study variables

Data were categorized into the following groups: demographic characteristics, injury characteristics, medical interventions, immunization management, and others. Demographic data collected for all patients included sex, age, occupation, and residential status (categorized as local or migrant population based on household registration information). Detailed injury information was recorded, including date of injury, location of injury, species of the injuring animal, exposure grade, exposure risk, wound site, number of wounds, history of vaccination, and whether passive immunizing agents were administered. The patient’s history of prior tetanus vaccination and whether they received tetanus immunoglobulin at Zhejiang Hospital were also documented. The exposure grade was classified according to the World Health Organization (WHO) recommended guidelines. For animal-induced injuries, wound cleanliness was categorized as follows: Class I wounds (clean wounds), Class II wounds (unclean wounds), and Class III wounds (contaminated wounds). Wound risk classification was defined as: Class I exposure as low risk, and Class II & III exposures as high risk. Intervention and immunoprophylactic measures implemented for the attending animal-induced injury patients were extracted from the hospital’s electronic medical record system. Monthly average temperature data for Hangzhou City, Zhejiang Province, from February 1, 2024, to January 31, 2025, were obtained from the National Centers for Environmental Information of the National Oceanic and Atmospheric Administration. Months were categorized as warm or cold based on the average temperature.

### 2.4 Statistical analysis

Data analysis and graphing were performed using SPSS 26.0 and Prism 9.5.0 statistical software. Measurement data are presented as mean ± standard deviation (X ± S) and were compared using the t-test. Categorical data are presented as rates (percentages, %) and were compared using the chi-square (χ^2^) test. For measurement data that were not normally distributed, values are expressed as the median (interquartile range) [M (P25, P75)], and comparisons between two groups were made using the Mann–Whitney U test. A two-sided *p* value < 0.05 was considered statistically significant.

### 2.5 Ethics approval and consent to participate

The study protocol adhered to the Declaration of Helsinki. The protocol for this retrospective study was reviewed and approved by the Ethics Review Committee of Zhejiang Hospital (approval no. ZJHIRB-2025-098 K) on June 13, 2025. This study is also registered with the Chinese Clinical Trial Registry (registration no. ChiCTR2500108189). Given the retrospective design and the exclusive use of fully anonymized patient data, the Ethics Review Committee waived the requirement for written informed consent.

## 3 Results

### 3.1 Demographic characteristics

**Figure 1:**
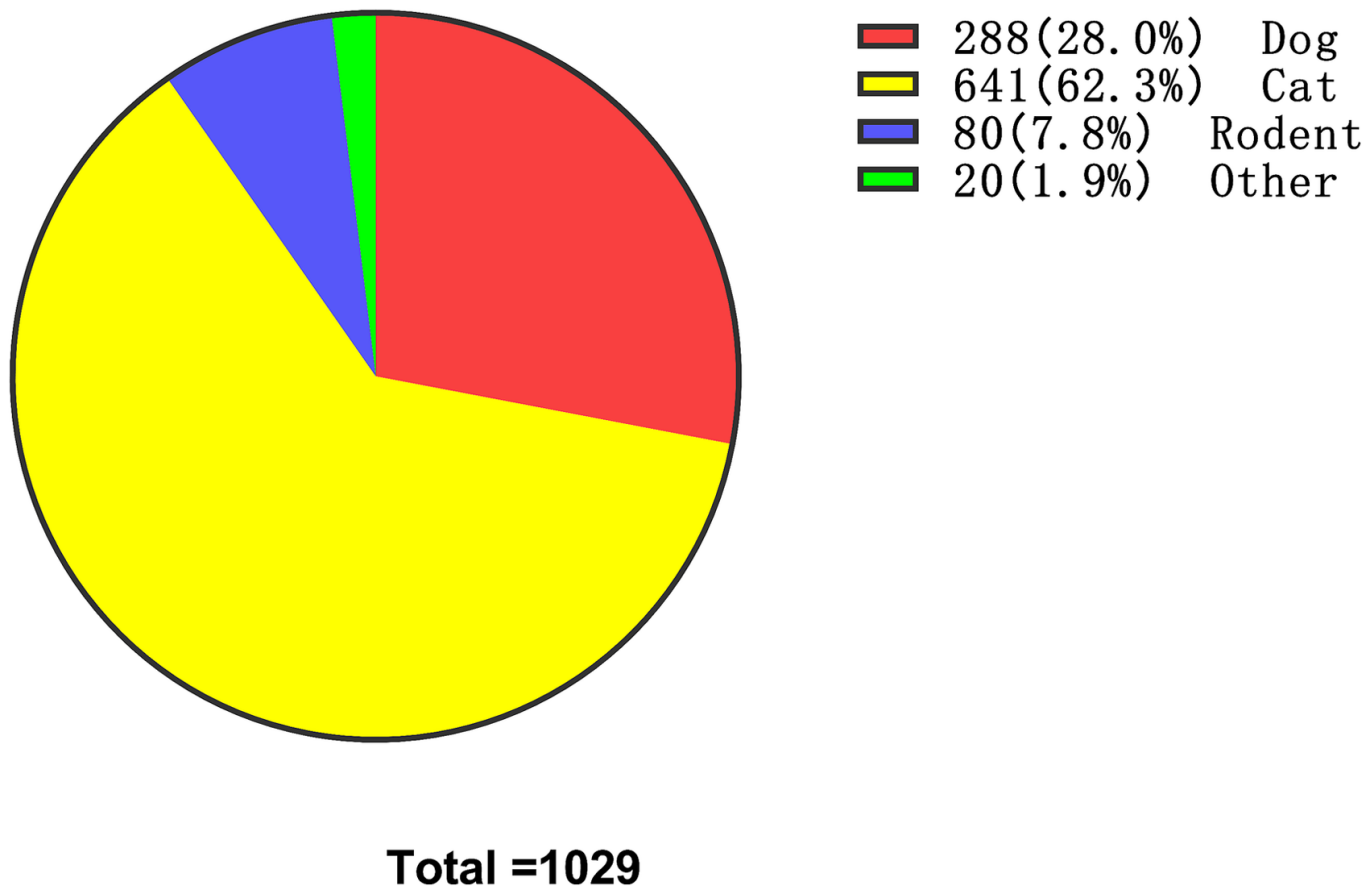
Proportion of animal-induced injury patients.

From February 2024 to January 2025, a total of 3,174 patients who received tetanus vaccination at the Emergency Department of Zhejiang Hospital were initially identified. After applying the exclusion criteria (incomplete clinical data or refusal of prophylactic immunization), 2,825 patients were included in the final analysis. Among the included patients, animal-induced injuries accounted for 1,029 cases (36.4%), traffic accident injuries for 943 cases (33.4%), cutting injuries for 374 cases (13.2%), blunt force injuries for 242 cases (8.6%), and other causes (e.g., falls) for 237 cases (8.4%). Gender distribution: Males predominated in all trauma categories except animal-induced injuries. In contrast, females were the majority in animal-induced injuries (53.3%). Age distribution: The 25–35 years age group was the largest across most injury categories, comprising 48.3% of traffic accident injuries, 53.7% of blunt force injuries, 44.6% of animal-induced injuries, and 37.4% of other injuries. Geographic origin: Migrants from other provinces constituted the majority in all trauma categories. In contrast, local residents were the majority only among patients with animal-induced injuries (57.9%). Occupation distribution: Employees/workers were the predominant occupational group across all injury categories (Table 1; Figure 1).

**Table 1:** Demographic characteristics of patients with injuries.

Characteristic	Animal-induced injuries	Traffic accident injuries	Cutting injuries	Blunt force injuries	Other (e.g., falls, etc.)
Gender [*n* (%)]					
Male	480 (46.7)	576 (61.1)	218 (58.3)	142 (58.7)	145 (60.9)
Female	548 (53.3)	367 (38.9)	156 (41.7)	100 (41.3)	93 (39.1)
Age [*n* (%)]					
<11 years	17 (1.7)	6 (0.6)	9 (2.4)	5 (2.1)	9 (3.8)
12–24 years	323 (31.4)	246 (26.1)	155 (41.4)	52 (21.5)	98 (41.2)
25–35 years	458 (44.6)	455 (48.3)	110 (29.4)	130 (53.7)	89 (37.4)
36–44 years	74 (7.2)	117 (12.4)	24 (6.4)	22 (9.1)	14 (5.9)
45–64 years	120 (11.7)	88 (9.3)	57 (15.2)	27 (11.2)	21 (8.8)
>65 years	36 (3.5)	31 (3.3)	19 (5.1)	6 (2.5)	7 (2.9)
Geographic origin [*n* (%)]					
Local residents	595 (57.9)	294 (31.2)	122 (32.6)	84 (34.7)	94 (39.1)
Migrants from other provinces	433 (42.1)	649 (68.8)	252 (67.4)	158 (65.3)	145 (60.9)
Occupation [*n* (%)]					
Infant/toddler	0 (0)	0 (0)	4 (1.1)	1 (0.4)	1 (0.)
Student	208 (20.2)	160 (17.0)	111 (29.7)	36 (14.9)	71 (29.8)
Employee/worker	697 (67.8)	705 (74.8)	211 (56.4)	185 (76.4)	144 (60.5)
Retired	51 (5.0)	44 (4.7)	27 (7.2)	8 (3.3)	11 (4.6)
Other	72 (7.0)	34 (3.6)	21 (5.6)	12 (5.0)	11 (4.6)

### 3.2 Injury characteristics

**Figure 2:**
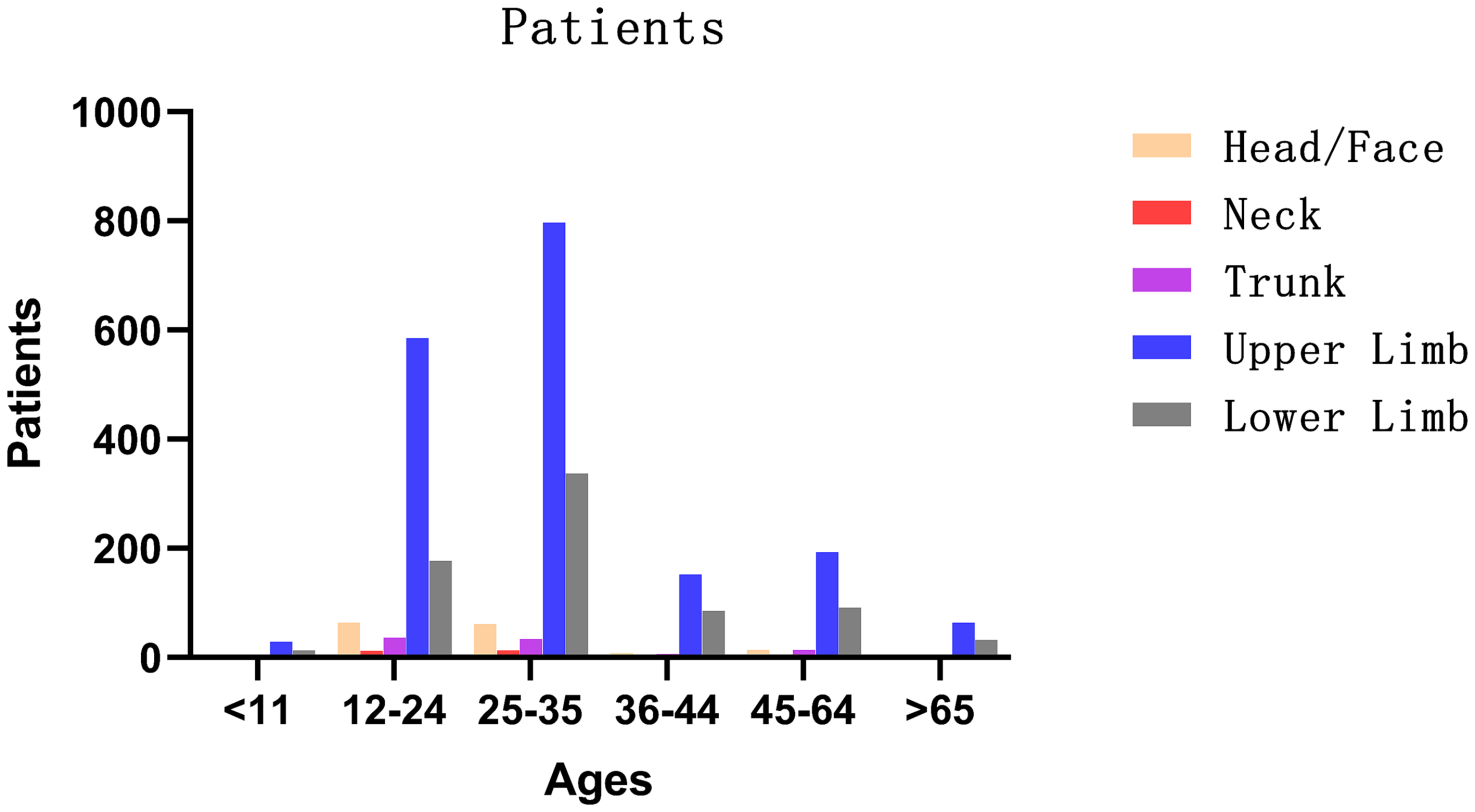
Number of patients with injury by age group.

Location of injury: Among patients with animal-induced injuries, the vast majority occurred indoors (841 cases, 81.8%). For cutting injuries, 154 cases (41.2%) occurred indoors and 220 cases (58.8%) outdoors. Wound site: The upper limb was the most common injury site across all categories, accounting for 74.1% of animal-induced injuries, 59.4% of traffic accident injuries, 59.7% of cutting injuries, 58.2% of blunt force injuries, and 56.7% of other injuries. Wound risk classification: High-risk wounds were observed in the majority of patients across all categories, with the highest proportion in animal-induced injuries (1,018 cases, 98.9%). Administration of passive immunizing agents: The use of passive immunizing agents was generally low across all categories. Standardized prescription: Overall, standardized prescriptions-defined as management in accordance with the 2024 Chinese guidelines for non-neonatal tetanus-were issued for 2,766 patients, yielding a standardization rate of 97.9%. Number of wounds: Single wounds were predominant in all injury categories (Table 2; Figure 2).

**Table 2:** Injury characteristics of patients with injuries.

Characteristic	Animal-induced injuries	Traffic accident injuries	Cutting injuries	Blunt force injuries	Other (e.g., falls, etc.)
Location of injury [*n* (%)]					
Indoors	841 (81.8)	0 (0)	154 (41.2)	115 (47.5)	107 (45.0)
Outdoors	187 (182)	943 (100)	220 (58.8)	127 (52.5)	131 (55.0)
Wound site [*n* (%)]					
Head/Face	33 (3.2)	64 (6.8)	29 (7.8)	14 (5.8)	14 (5.9)
Neck	2 (0.2)	13 (1.4)	3 (0.8)	4 (1.7)	4 (1.7)
Trunk	18 (1.7)	41 (4.3)	7 (1.9)	8 (3.3)	16 (6.7)
Upper limb	761 (74.1)	560 (59.4)	223 (59.7)	141 (58.2)	135 (56.7)
Lower limb	214 (20.8)	265 (28.1)	112 (15.2)	75 (31)	69 (29.0)
Number of wounds [*n* (%)]					
Single	915 (89.0)	911 (96.6)	366 (97.9)	238 (98.3)	231 (97.1)
Multiple	113 (11.0)	32 (3.4)	8 (2.1)	4 (1.7)	7 (2.9)
Risk [*n* (%)]					
Low risk	11 (1.1)	85 (9.0)	20 (5.3)	19 (7.9)	28 (11.8)
High risk	1,018 (98.9)	858 (91.0)	354 (94.7)	223 (92.1)	209 (88.2)
Administration of passive immunizing agents [*n* (%)]					
Administered	107 (10.4)	31 (3.3)	21 (5.6)	10 (4.1)	5 (2.1)
Not administered	922 (89.6)	912 (96.7)	353 (94.4)	232 (95.9)	232 (97.9)
Standardized Prescription [*n* (%)]	2,766 (97.9)				

Standardized prescription refers to the proportion of patients who received management in accordance with clinical guidelines at the Emergency Department of Zhejiang Hospital.

### 3.3 Epidemiological and seasonal characteristics

**Figure 3:**
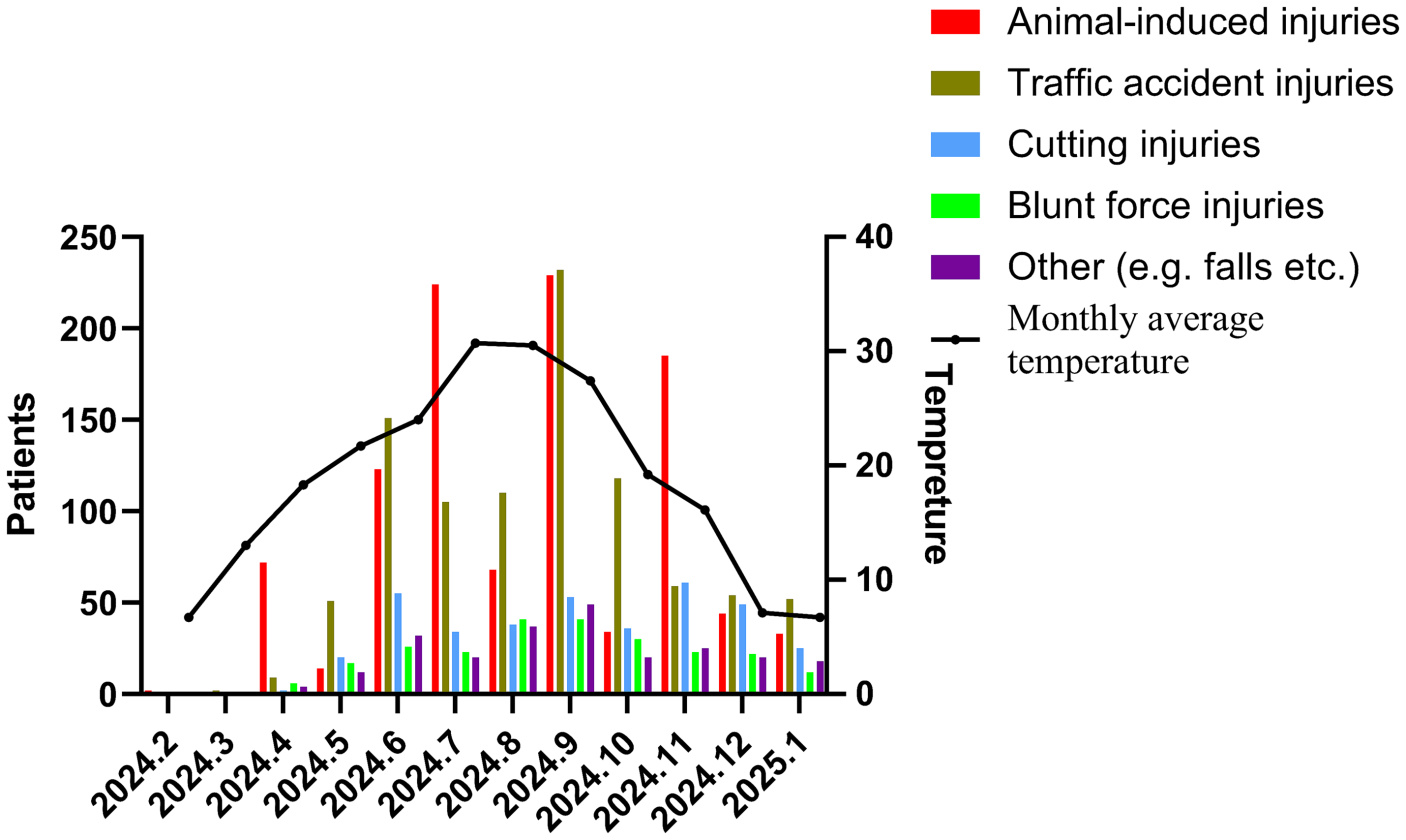
Number of individuals receiving tetanus vaccination by month.

Tetanus vaccine prescriptions were recorded in all study months, with the prescription rate showing a monthly increasing trend. The number of animal-induced injuries during warm months was significantly higher than that of other traumas occurring in the same period, and the difference was statistically significant (*p* < 0.01). Among patients with other traumas, the proportion of indoor injuries occurring in warm months was significantly higher than that of outdoor injuries occurring in warm months (*p* < 0.01, Table 3). In contrast, for animal-induced injuries, the proportional distribution of indoor and outdoor injuries between cold and warm months was similar, with no statistically significant difference (*p* > 0.05). Regarding the species responsible for animal-induced injuries, cats were the most common source, accounting for 641 cases (62.3%), followed by dogs (Figures 1, 3).

**Table 3:** Comparison of the number of patients with animal-induced injuries and other traumas during cold and warm months.

Category	Cold months	Warm months	χ^2^ value	*p* value
Animal-induced injury patients [*n* (%)]	186 (18.1)	843 (81.9)		
Other trauma patients [*n* (%)]	445 (24.8)	1,351 (75.2)	16.97	<0.01
Animal-induced Injuries occurred indoors [*n* (%)]	25 (3.1)	794 (96.9)		
Animal-induced Injuries occurred outdoors [*n* (%)]	420 (43.0)	557 (57.0)	381.25	<0.01
Other trauma Injuries occurred indoors [*n* (%)]	148 (17.6)	694 (82.4)		
Other trauma Injuries occurred outdoors [*n* (%)]	38 (20.3)	149 (79.7)	0.78	0.38

## 4 Discussion

Tetanus is an extremely severe and potentially fatal disease caused by *Clostridium tetani*. In China, thanks to the implementation and improvement of planned immunization programs, the number of tetanus cases has declined significantly compared to previous years. However, a considerable gap remains compared to developed countries. For instance, data from Canada show that only 155 tetanus cases were recorded in hospitals between 1995 and 2019, with 10 deaths (13). A crucial contributing factor is the standardized use of active and passive immunizing agents. China witnesses over 200 million trauma cases annually (14). Traditionally, tetanus prevention in trauma patients relied solely on tetanus immunoglobulins or tetanus antitoxins rather than the tetanus vaccine. Studies indicate that the annual usage of tetanus antitoxin in China ranges from 40 to 50 million doses (15), and the use of tetanus immunoglobulin reached 3.77 million patient-doses in 2016 alone (16). The non-standardized use of passive immunizing agents leads to a waste of medical resources and inappropriate management. Research has shown that *C. tetani* can persist in trauma patients for extended periods. Tetanus antibodies induced by active immunization achieve higher and more sustained titers compared to those from passive immunization (17, 18), with fewer side effects. In summary, the standardized combined use of tetanus active and passive immunizing agents is demonstrably superior to the use of passive agents alone.

In this study, from February 2024 to January 2025, all trauma and animal-induced injury patients who received tetanus vaccination at the Emergency Department of Zhejiang Hospital were initially identified from the electronic medical record system. Patients were excluded if: (1) any essential clinical information was missing, or (2) they refused to receive prophylactic immunization. After applying these criteria, a total of 2,825 patients were included in the final analysis. A total of 1,029 patients with animal-induced injuries and 1,796 patients with other traumas received tetanus vaccination in our hospital’s emergency department. The number of tetanus vaccinations in February and March 2024 was notably lower than in other months, while a distinct peak was observed from June to September 2024. This suggests that public awareness campaigns for tetanus vaccination should be intensified during summer and autumn. Regarding demographic characteristics, most patients with other traumas were migrants from other provinces, whereas most animal-induced injury patients were local residents. This indicates a need to target animal injury prevention education towards the local population and trauma prevention education towards the migrant population. Notably, other trauma patients were concentrated in the 19–37 age group, and animal-induced injury patients in the 19–34 age group, largely overlapping. Within this age range, students and employees/workers constituted the majority, making them key target groups for educational efforts.

Regarding injury characteristics, indoor and outdoor injuries occurred at similar proportions among other trauma patients. In contrast, animal-induced injuries occurred predominantly indoors. High-risk wounds were significantly prevalent in both groups. Given that cats were the leading cause of animal injuries, it is reasonable to infer that most animal-induced injuries occur indoors due to interactions with domestic cats. This pattern differs from international reports (19, 20) and reflects regional preferences, as urban Chinese residents are more likely to keep cats than dogs as pets. This highlights the need for targeted education on animal injury and tetanus prevention for pet owners, particularly cat owners.

The number of tetanus vaccinations was very low in February and March 2024, began to increase in April, and was significantly higher during the summer and autumn months (June to September 2024). This trend aligns with other studies on trauma and animal injuries (21, 22). However, this increase is not solely attributable to the higher incidence of injuries in warmer seasons. Our research initiated tetanus vaccination services in January 2024. Initially, there was variability among surgeons in updating their treatment concepts, and some patients refused tetanus vaccination, insisting only on tetanus immunoglobulin. Consequently, many cases involved the standalone use of tetanus immunoglobulin rather than the standardized protocol of assessment followed by appropriate use of vaccine and/or immunoglobulin. Following intensified hospital training on the standardized management of non-neonatal tetanus and the release of China’s 2024 guideline for non-neonatal tetanus diagnosis and treatment in October (12), prevention practices became more standardized, leading to a marked increase in tetanus vaccinations. The assessment-based formulation of tetanus prevention plans gradually replaced the non-assessed, routine use of tetanus immunoglobulin. Overall, during the first year of implementing tetanus vaccination, our research achieved a standardized prescription rate of 97.9%. However, non-standard prescriptions persisted. Review of these cases revealed that most resulted from patient or family insistence on or refusal of passive immunizing agents despite medical advice, or from healthcare providers adhering to traditional practices or not strictly following the 2024 national guideline. Continued public education and healthcare worker training are therefore essential.

The age distribution of injured patients shows a concentration in the 12–35 age group, predominantly comprising employees/workers. Considering China’s immunization policy, the nationwide planned immunization program was considered fully implemented by 1989. Therefore, animal-induced and other trauma patients over 35 years old seen between February 2024 and January 2025 had not completed their primary tetanus vaccination series. All these patients had high-risk wounds. Other countries have adult catch-up vaccination programs for such individuals (23). Similarly, we should implement measures to complete the tetanus vaccination series for these adult patients.

This study also collected monthly average temperatures for Hangzhou, Zhejiang Province, from February 2024 to January 2025. Based on the median monthly temperature, months were categorized as warmor cold. The number of animal-induced injuries during warm months was significantly higher than that of other traumas during the same period. Among other trauma patients, the proportion of indoor injuries occurring in warm months was significantly higher than that of outdoor injuries occurring in warm months. This suggests that other trauma patients were more likely to sustain indoor injuries during warm months, possibly due to reduced outdoor activities in hot weather. The higher incidence of animal-induced injuries in warm months may be attributed to greater skin exposure.

### 4.1 Strengths

1. The data-source hospital is one of the first in Zhejiang Province to offer tetanus vaccination and a primary institution in Hangzhou for non-neonatal tetanus immunization, providing extensive data on trauma and animal-induced injury patients from a relatively reliable emergency electronic medical record system. 2. As mentioned, tetanus vaccination for animal-induced injury patients has gained attention in China only recently, whereas previously the focus was solely on rabies vaccine and tetanus passive immunizing agents. Thus, the data presented here are relatively novel. 3. A key strength of our study is the ability to track the completion of the full post-exposure prophylaxis course for rabies, a metric often unavailable in studies limited to data from a single visit.

### 4.2 Limitations

1. The data originate from the emergency department of a single hospital serving a population equivalent to a medium-sized city (approximately 1 million). Therefore, the findings may not be fully representative of trauma and animal injury patterns in broader regions (e.g., provincial or national levels). 2. Due to the lack of official, age- and sex-stratified population data for our emergency department’s catchment area, we could not perform population-standardized adjustments in this study.

In conclusion, a gap persists between tetanus prevention in China and that in developed countries, significantly attributable to the non-standardized application of active and passive immunizing agents. Outdated practices among some healthcare workers, stemming from a lack of updated knowledge, necessitate enhanced training and public education. Based on our research’s data analysis, targeted education should focus on residential and urban communities, particularly students, migrant workers, and pet owners, with intensified efforts during the warmer summer and autumn months.

## Data Availability

The raw data supporting the conclusions of this article will be made available by the authors, without undue reservation.
